# Polyenic Antibiotics and Other Antifungal Compounds Produced by Hemolytic *Streptomyces* Species

**DOI:** 10.3390/ijms232315045

**Published:** 2022-11-30

**Authors:** Jan Bobek, Eliška Filipová, Natalie Bergman, Matouš Čihák, Miroslav Petříček, Ana Catalina Lara, Vaclav Kristufek, Melinda Megyes, Theresa Wurzer, Alica Chroňáková, Kateřina Petříčková

**Affiliations:** 1 Institute of Immunology and Microbiology, 1st Faculty of Medicine, Charles University, Studničkova 7, 128 00 Prague, Czech Republic; 2 Faculty of Science, Jan Evangelista Purkyně University in Ústí nad Labem, České mládeže 8, 400 96 Ústí nad Labem, Czech Republic; 3 Contipro a.s., Dolní Dobrouč 401, 561 02 Dolní Dobrouč, Czech Republic; 4 Institute of Soil Biology and Biogeochemistry, Biology Centre of the Czech Academy of Sciences, Na Sádkách 7, 370 05 České Budějovice, Czech Republic; 5 Doctoral School of Environmental Sciences, ELTE Eötvös Loránd University, 1117 Budapest, Hungary; 6 Faculty of Science, University of South Bohemia in České Budějovice, Branišovská 31, 370 05 České Budějovice, Czech Republic

**Keywords:** *Actinomycetales*, secondary metabolites, polyene antibiotics, *Streptomyces*, hemolysis, symbiosis, soil ecosystem

## Abstract

*Streptomyces* are of great interest in the pharmaceutical industry as they produce a plethora of secondary metabolites that act as antibacterial and antifungal agents. They may thrive on their own in the soil, or associate with other organisms, such as plants or invertebrates. Some soil-derived strains exhibit hemolytic properties when cultivated on blood agar, raising the question of whether hemolysis could be a virulence factor of the bacteria. In this work we examined hemolytic compound production in 23 β-hemolytic *Streptomyces* isolates; of these 12 were soil-derived, 10 were arthropod-associated, and 1 was plant-associated. An additional human-associated *S.* sp. TR1341 served as a control. Mass spectrometry analysis suggested synthesis of polyene molecules responsible for the hemolysis: candicidins, filipins, strevertene A, tetrafungin, and tetrin A, as well as four novel polyene compounds (denoted here as polyene A, B, C, and D) in individual liquid cultures or paired co-cultures. The non-polyene antifungal compounds actiphenol and surugamide A were also identified. The findings indicate that the ability of *Streptomyces* to produce cytolytic compounds (here manifested by hemolysis on blood agar) is an intrinsic feature of the bacteria in the soil environment and could even serve as a virulence factor when colonizing available host organisms. Additionally, a literature review of polyenes and non-polyene hemolytic metabolites produced by *Streptomyces* is presented.

## 1. Introduction

### 1.1. The Role of Hemolysis in Streptomyces’ Interactions

*Streptomyces* produce a large variety of secondary metabolites (SM) that correspond to their environmental needs [1], of which antibacterial and antifungal agents are of utmost importance [2]. The chemical diversity of the SMs produced by *Streptomyces* species has most likely evolved as a direct result of their interactions with other organisms. *Streptomyces’* interactions with plants and animals can be parasitic, as is the case of potato scab-causing streptomycetes, which infect the plant tuberosphere [3], or *S. somaliensis* and other strains that infect humans [4]. However, in most cases, they are beneficial and growth- promoting organisms. Streptomycetes form numerous mutualistic relationships with invertebrates and plants [5], and they protect their hosts against infection using antibiotics and antifungals.

Hemolysis—the rupture of erythrocytes—is a virulence factor of many pathogens with *E. coli, Streptococci, Vibrio,* and *Staphylococcus aureus* being some prominent examples [6]. The activity of hemolysins, however, is not restricted to erythrocytes only; hemolysins, by acting on cellular membranes, can also damage other eukaryotic cells [6,7]. Hemolysins are mostly lytic proteins—enzymes or channel-forming porins [8] or, rarely, polyene compounds (also referred to as polyene antibiotics or polyene antimycotics) [9].

### 1.2. Polyenes and Non-Polyene Hemolytic Metabolites: A Literature Review

Polyenes are poly-unsaturated compounds with linear or cyclic structures. Cyclic polyene antibiotics belong to the macrolide class of SMs, representing a large and variable group of antibiotics produced mostly by *Streptomyces*. They have a macrolactone ring to which typically two sugars and one amino sugar are attached [10]. Antibiotics in the polyene class possess a macrocyclic ring of carbon atoms closed by lactonization; the polyene group has, in addition, a series of conjugated double bonds of various length.

We searched the literature extensively to review mostly actinomycete-derived polyenes identified so far. They are listed in groups based on their structures together with their chemical formulas, molecular weight (MW), and assessed activities (Table 1); the representative structures are shown in Figure S1. Selected non-polyene, human cells-targeting metabolites included in the study are placed in Table 2. The primary selection criterion was the origin of compounds in streptomycetes or related bacteria and their reported activity towards eukaryotic cells. The majority of the compounds were discovered more than 50 years ago, therefore the data often lack sufficient structure determination and complex activity screenings. Crucial structural characteristics include a combination of a hydrophobic polyene region with a hydrophilic polyol part, often glycosylated, which enables the molecules to enter the cytoplasmic membranes of various organisms, exhibiting either irreversible destruction of the membrane or transient and reversible channel formation (e.g., in pentaenes or heptaenes, respectively). They form complexes, in which the polyene chain faces the lipid environment and the polyol chain is oriented towards the aqueous environment in the interior of the pores [11]. Polyenes often form complexes with sterols and exhibit variable specificities to ergosterols and cholesterols [12]. These traits strongly influence their cytotoxicity and, subsequently, their possible medical application.

Almost all polyene compounds have been identified due to their antifungal activity [11] and some have been shown to possess other bioactivities, such as antibacterial (often targeting cell-wall lacking bacteria), antiparasitic (anti-*Trichomonas* activity has been reported most frequently, implying the compounds may find use in the treatment of combined vaginal infections), cytolytic, and anti-cancer activities.

Most of polyene antibiotics, including filipin and candicidin compounds with larger rings, exhibit hemolytic properties [89]. Filipin forms large aggregates within the erythrocyte membrane that render it permeable [90]. Other polyenes, however, impair plasma membranes by direct binding to ergosterol, as is the case of natamycin [91] or amphotericin [92]. All these polyene compounds are fungicidal and those with lower toxicity are used in medicine. For example, amphotericin B, natamycin and nystatin A1 are used in antifungal and antiprotozoal medications [90].

### 1.3. Hemolysis as a Virulence Factor

Whilst SM production in *Actinobacteria* has been extensively studied [2,69], the impact of hemolytic metabolites has not received much attention so far. As hemolysis can be considered a virulence factor [93,94], it may well be viewed as one of the adaptations that the bacteria employ to compete with other organisms in their environment. This theory is further supported by the example of the streptomycete strain *S.* sp. TR1341, extracted from the lungs of a senior male patient with relapsing bronchopneumonia, whose taxonomy indicates a distance from plant and human pathogenic strains [89]. It has been demonstrated that *S.* sp. TR1341 possesses a filipin biosynthetic gene cluster responsible for the bacteria’s hemolytic capabilities [89]. 

The β-hemolytic activity is not exclusively related to human-associated strains, but also occurs in soil-dwelling strains. About half of soil-derived *Streptomyces* strains exhibit β-hemolytic activity, whereas three out of four clinical *Streptomyces* isolates are β-hemolytic, according to Wurzer [95]. The soil-derived strains exhibiting β-hemolysis were collected (BCCO strains) and used here to search for the β-hemolytic compound production. The search was focused on polyene antibiotics as their role as hemolysins has not yet been systematically studied in *Streptomyces*. In addition, the 16S rRNA phylogeny of the BCCO strains used in this study was compared to that of well-known polyene producers.

## 2. Results

### 2.1. Sequencing Data and Phylogenetic Tree

**Figure 1:**
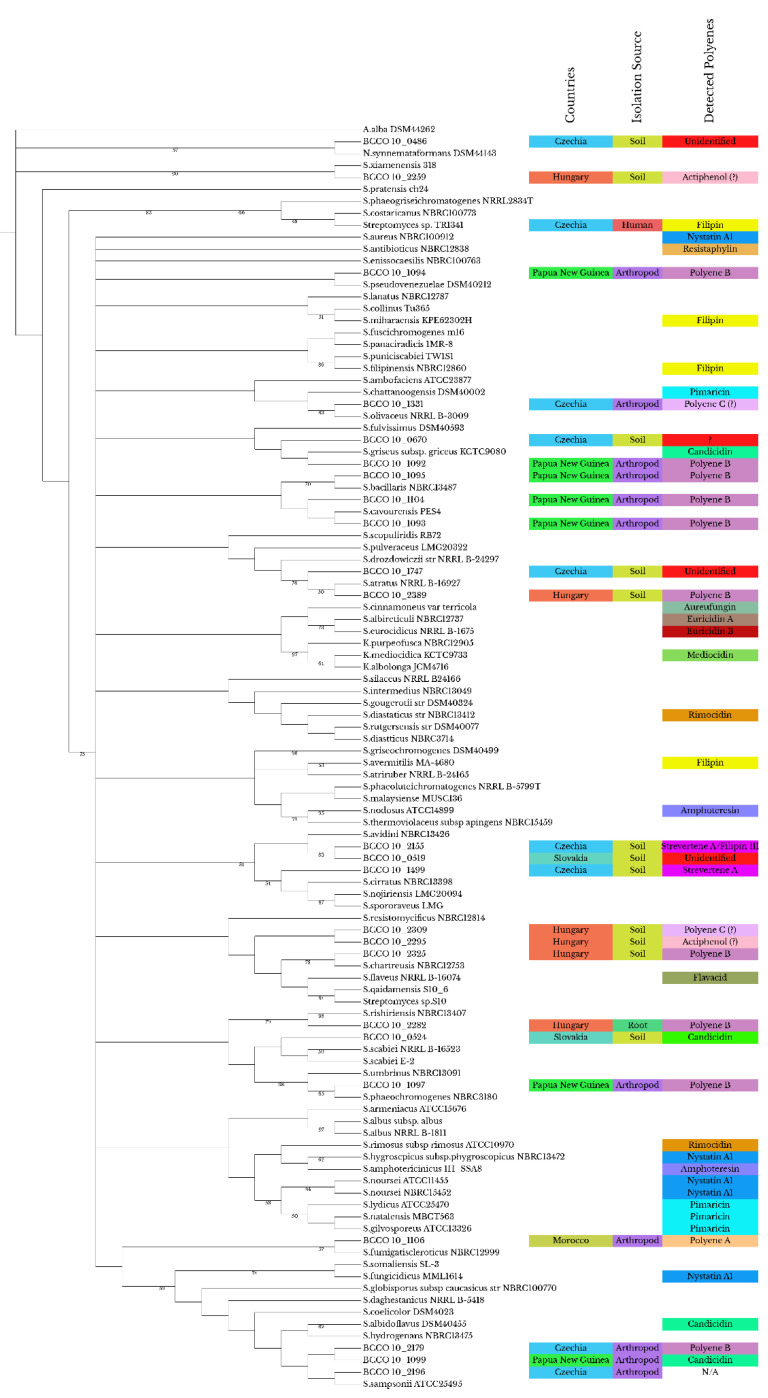
**Phylogeny of *Streptomyces* strains involved in the study inferred from 16S rRNA gene similarity**. Color codes represent the type of a polyene produced by the strain, the isolation source, and the country of origin. Only bootstraps above 50 are shown. The question mark indicates metabolites detected in a co-culture. The sequences of type strains closely related to the sequences of known polyene producers and BCCO strains were included to support topography of phylogenetic tree. The strains were selected according to Labeda et al. [96]. Abbreviations: A.–*Amycolatopsis*, N.–*Nocardioides*, S.–*Streptomycces*.

According to the 16S rRNA-encoding gene similarities, 22 BCCO strains with β-hemolytic activities clustered with other *Streptomyces* and one strain with *Nocardioides* (BCCO 10_0486). Phylogenetic analysis showed no clear association between phylogeny and the production of particular polyene compounds (Figure 1); the clustering seems to correlate more with the isolation source (arthropod or soil) or the country of origin. However, there are a few clusters that contain strains of various origins (country/source): (i) BCCO 10_1099 (Papua New Guinea/ambrosia beetle) and BCCO 10_2196 (Czechia/millipede) clustered together with *S. albidoflavus* DSM40455; (ii) BCCO 10_0670 (Czechia/soil) and BCCO 10_1092 (Papua New Guinea/ambrosia beetle) clustered together with *S. griseus* subsp. *griseus* KCTC9080; and (iii) BCCO 10_1747 (Czechia/soil) and BCCO 10_2389 (Hungary/soil) clustered as *S. drozdowiczii* NRRL-B-24297. 

Apparently, filipin, candicidin, nystatin A1, pimaricin, and actiphenol can be produced by strains from different phylogenetic groups. Our analysis suggests that strains with high phylogenetic relatedness and originating from the same habitat and country produce either the same polyene B (strains associated with ambrosia beetle from Papua New Guinea: BCCO 10_1093, 10_1095, 10_1104) or different compounds (soil strains from Hungary: BCCO 10_2295, 10_2309, 10_2325), as can be seen in our results below.

### 2.2. Characteristics of Morphological Differentiation and Hemolytic Activities in the Strains

Investigated strains were streaked on blood agar plates, and after 120 h of cultivation, pictures of growing mycelia and hemolytic zones were taken from the top and bottom sides of the dish. All lab-tested strains expressed β-hemolytic activity when grown on blood agar (Table S2).

### 2.3. Gamma-Butyrolactone-Induced Polyene Production

*Gamma*-butyrolactone (GBL) is an activator of SM production in *Streptomyces.* To test its capacity to induce polyene production, we inoculated a strain of *Streptomyces* as one line on the blood agar. Five microliters of GBL solution (ReagentPlus, ≥99%, Sigma-Aldrich Co. St. Louis, MO, USA) were dropped on a sterile filter paper strip which was then inserted perpendicularly to the line of *Streptomyces*. An increase in the hemolytic zone was observed after O/N cultivation on the cross-sections with the GBL-soaked paper strip in BCCO 10_0524, BCCO 10_0670, BCCO 10_1093, BCCO 10_1747, BCCO 10_2179, and BCCO 10_2389 (Figure S2). Remaining strains did not reveal any phenotypic difference in the presence of GBL.

### 2.4. Hemolytic Activity of Ethyl Acetate Extracts

**Figure 2:**
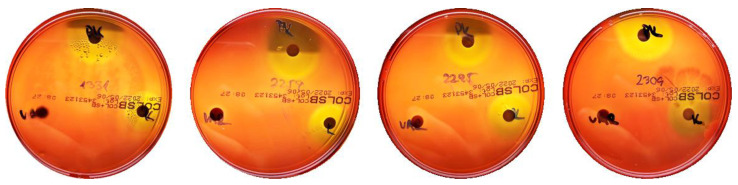
Hemolytic activity of supernatants after proteinase K treatment or boiling. Cell-free supernatants from 72-h-old cultures of strains BCCO 10_1331, BCCO 10_2259, BCCO 10_2295, BCCO 10_2309 (listed from left) were treated by proteinase K (upper discs on each plate) or were boiled (discs on the left of each plate), or no additional treatment have been performed (control discs on the right side of each plate).

To determine whether SMs produced by *Streptomyces* spp. into the medium could cause hemolysis, the culture supernatants were subjected to ethyl acetate extraction. A similar approach has already been used [89] to demonstrate that the polyene compound filipin is the only compound responsible for the hemolytic activity of their tested strain. The crude extracts (5 μL) were dropped on blood agar plates and incubated for 3 days with 5 μL of chloroform in the middle as a negative control (Figure S3). These experiments were performed in duplicate. Hemolytic activity was spotted on the blood agar for BCCO 10_1093, BCCO 10_1094, BCCO 10_1095, BCCO 10_1106, and BCCO 10_1499 strains. No hemolytic zone was observed in samples isolated from the BCCO 10_1099 strain and from the negative control. Nevertheless, the ethyl acetate extraction procedure led to a considerable reduction in the hemolytic activity when compared to hemolytic zones produced by the cell-free supernatants shown in Figure 2 (see below).

### 2.5. LC-MS Analysis of the Supernatant Extracts

After cultivation in liquid medium with or without blood, SPE was performed using the supernatants, followed by LC-MS. The presence of the polyenes listed in Table 1 was assayed in the metabolic extracts of selected beta-hemolytic streptomycete strains. The length of the polyene part influences the typical three-peak UV-VIS spectrum of the compounds [42]. This was used as a clue to identify putative, so far uncharacterized, polyenes in some extracts. The LC-MS analysis results are summarized in Table S3. The metabolites found are also listed in Figure 1 and Table S2.

Out of the 23 β-hemolytic tested strains, known polyene substances were likely detected in 12 strains (Table S2). Of these, candicidins A1–A3 were detected in two strains (BCCO 10_0524, BCCO 10_1099). Strevertene A was detected in two strains (BCCO 10_1499, BCCO 10_2155) and filipin III in BCCO 10_2155. Tetrafungin and tetrin A were found in BCCO 10_2325, BCCO 10_1092, BCCO 10_1093, BCCO 10_1094, BCCO 10_1095, BCCO 10_1104, and BCCO 10_2179.

A new polyene (retention time (tR) = 9.42 min, [M+H]^+^ m/z = 745.4166, UV/VIS wavelength of maximum absorbance: (289 nm), 327 nm, 343 nm, 362 nm), designated here as polyene B, was likely detected in 10 strains (BCCO 10_1092, BCCO 10_1093, BCCO 10_1094, BCCO 10_1095, BCCO 10_1097, BCCO 10_1104, BCCO 10_2179, BCCO 10_2282, BCCO 10_2325, and BCCO 10_2389). Its absorption spectrum suggests the presence of a pentaene structure, and the production is often associated with the formation of tetrafungin and tetrin A compounds (Table S2). The second novel polyene compound (tR = 8.23 min, UV/VIS wavelength of maximum absorbance: 311 nm, 326 nm, 343 nm) was designated polyene A and was present only in the extract of BCCO 10_1106 strain. The third, polyene D, tR = 10.6 min, UV/VIS wavelength of maximum absorbance: 287 nm, 345 nm, 362 nm, 384 nm, most probably has hexaene or methylhexaene structure [42]. No polyene-like compound was detected in individual cultures of five β-hemolytic strains (BCCO 10_0670, BCCO 10_1331, BCCO 10_2259, BCCO 10_2295, BCCO 10_2309), in both 7% blood-containing and blood-free media.

Besides the above-described polyenes, the extracts were screened for the presence of non-polyene, human cells-targeting SMs (Table 2). Of these, we identified Surugamide A, a non-ribosomal peptide with cathepsin B-inhibitory activity [70], in BCCO 10_0524 and BCCO 10_1099, the two strains that also produced candicidin A1-A3. The latter strain belongs to the *S. albidoflavus* clade according to our phylogenetic tree. In a recent study, *S. albidoflavus* J11074 has also been reported as a surugamide producer during cultivation under stress conditions [68].

### 2.6. Selected Hemolytic Activity Is Likely Not Due to Lytic Proteins

Four β-hemolytic strains (BCCO 10_1331, BCCO 10_2259, BCCO 10_2295, BCCO 10_2309), in which no polyene metabolite, and no other metabolites with possible hemolytic activity, were detected following standard cultivation, were further tested to show whether their hemolytic activity could be caused by extracellular protein(s). Each cell-free supernatant from these four 72-h-old cultures was split into three thirds: one was treated by proteinase K (1 μg/mL) for 60 min at 37 °C (to degrade extracellular hemolytic proteins), the second was incubated in the same conditions without the enzyme (serving as a negative control in which hemolytic activity was expected), and the third sample was incubated at 100 °C for 5 min (we expected it to lose its hemolytic activity completely). All samples lost their hemolytic activity after boiling, but not after the proteinase K treatment, suggesting that the hemolytic activity is associated with the SMs production rather than hemolytic proteins [97] in the tested streptomycetes (Figure 2).

### 2.7. Hemolytic Compounds Identified in Paired Co-Cultures

Four β-hemolytic strains (BCCO 10_1331, BCCO 10_2259, BCCO 10_2295, BCCO 10_2309), in which no polyene compound was detected, nor was hemolytic activity unique for extracytoplasmic proteins in those strains (see Section 2.7), were subjected to paired co-cultures to promote the production of SMs in these strains. In the sample isolated from the paired co-culture [BCCO 10_1331 + BCCO 10_2309] we detected a fourth novel polyene-like compound (UV/VIS wavelength of maximum absorbance: 346 nm, 364 nm, and 386 nm; tR = 7.77 min, acquired [M+H]^+^ = 743.4131), here designated as polyene C, with a putative (methyl-)hexaene structure. The second paired co-culture [BCCO 10_2259 + BCCO 10_2295] revealed a known hemolytic compound, namely actiphenol (a non-polyene antibiotic with MW 275; tR = 4.65 min, identified pseudomolecular ion [M+H]^+^ = 276.1219 and after the loss of hydroxyl group as a water molecule 258.1144), with antifungal effects [82] (Table S3). We were not able to detect any compound in other paired co-cultures (BCCO 10_2259 + BCCO 10_2309; BCCO 10_2259 + BCCO 10_1331; BCCO 10_2295 + BCCO 10_2309; BCCO 10_2295 + BCCO 10_1331).

### 2.8. Inhibitory Activity of Streptomyces against Candida albicans or Filamentous Fungi

*C. albicans* and filamentous micromycetes were cultured with streptomycetes to verify that the hemolytic streptomycetes also possess antifungal properties. The strains of *Streptomyces* were inoculated in lines and cultivated for 72 h. Then, lines of *C. albicans* were added perpendicularly to those of *Streptomyces* and the culture was continued for an additional 24 h. Pictures of the results were taken and the inhibitory zone of *C. albicans* was measured (Table 3).

Subsequently, strains of *Streptomyces* were cultured with *Aspergillus niger, Aspergillus fumigatus, Fusarium* spp., and *Paecilomyces* spp. to test for the antifungal activity of the produced SMs. The results of this experiment are summarized in Table 4. Based on these results, we may briefly conclude that streptomycetes producing polyene B are, to some extent, able to inhibit the growth of *Paecilomyces* spp. and *Aspergillus niger*, whereas *Aspergillus fumigatus* was mostly resistant. *Fusarium* spp. did not grow in the proximity of some polyene B-producing species (BCCO 10_1092, BCCO 10_1093, BCCO 10_1094), whereas it grew in the proximity of others (BCCO 10_1095, BCCO 10_1097, BCCO 10_1104, BCCO 10_2282, and BCCO 10_2389). The polyene A-producing BCCO 10_1106 inhibited only the growth of *Paecilomyces* spp., as was also the case of BCCO 10_2259, the strain which probably produces actiphenol. Strain BCCO 10_2309, the probable producer of polyene C, inhibited the growth of *Paecilomyces* spp. and *Aspergillus niger* but not that of *Fusarium* spp. and *Aspergillus fumigatus*. The candicidin-producing strain BCCO 10_1099 inhibited the growth of *Aspergillus niger, Fusarium* spp., and *Paecilomyces* spp., but it had no impact on that of *Aspergillus fumigatus*.

## 3. Discussion

Hemolytic capabilities might be beneficial to lyse eukaryotic cells in any streptomycete habitat, either to kill competitors or to obtain nutrients from their lysed cells. They might secondarily protect the streptomycete plant or animal symbionts against mycotic infections.

The SMs produced by streptomycetes traditionally receive considerable attention because of their promising and often successful clinical application [98]. Their hemolytic activities (or, in a broader sense, cytolytic activity, i.e., their ability to lyse eukaryotic cells) have, however, largely been overlooked, as the organisms are generally considered non-pathogenic. Nevertheless, hemolysis remains a natural part of the secondary metabolism of a substantial number of streptomycetes [90]. Recent studies have suggested that hemolysis may serve as a virulence factor [99,100], with filipin produced by *Streptomyces* sp. TR1341 as an example [89]. Cytolytic SMs may also provide the producer with the ability to acquire larger variability of substrates. Next, we cannot exclude the role of some hemolytic metabolites in the streptomycete programmed cell death, as has also been reported for other cytotoxins acting on DNA [101,102]; though the effect of polyenes in sterol-free bacterial membranes is much less severe [103].

To gain a better understanding of the role hemolysis might play in the soil ecosystem, we used streptomycete strains isolated from various environments for this study—12 isolates directly from the soil, 10 isolates from the bodies of invertebrates, such as ambrosia beetles (*Hadrodemius globus, Dinoplatypus pallidus, Xyleborus perforans, Diapus pussilimus, Crossotarsus mniszechi, Hypoborus ficus*) and millipedes (*Archispirostreptus gigas, Telodeinopus aoutii*), and 1 strain from plant roots (*Zea mays*). Ambrosia beetles live in symbiosis with ambrosia fungi to cover their nutritional needs and to create so-called fungal gardens. As such, they have developed a defensive symbiosis with actinomycetes, especially *Streptomyces* to protect themselves against fungi [104]. As the diversity of ambrosia beetles is huge (more than 3200 species), and ambrosia fungi are host-specific [105], it can be assumed that many different actinobacteria and their as yet unexplored secondary metabolites may be involved in defensive symbiosis in a particular species.

Our results indicate that a number of streptomycetes that live symbiotically or parasitically with invertebrates and plants produce polyenes as well as those living freely in soil. Interestingly, both beetle- and millipede-derived strains produce similar compounds, although the expected ecological functions differ: the defensive symbiosis of streptomycetes with ambrosia beetles is a well-known phenomenon [104]. On the other hand, there are no data about the symbiotic relationship of intestinal streptomycetes in millipedes. Their interaction may start incidentally, as a transient colonization. However, the fact that the same antifungal compounds produced in the beetles are produced in the millipedes may suggest a much tighter relationship, linked with the prevention of fungal pathogens growth in the host body.

To demonstrate a particular clash between streptomycetes and fungi, we performed a co-culture experiment using a yeast *C. albicans* (Table 3), and four different ascomycetes: *Aspergillus niger, Aspergillus fumigatus, Fusarium* spp., and *Paecilomyces* spp. (Table 4). When both organisms were inoculated at the same time, no inhibitory zones were observed, probably due to the time required for the onset of SM synthesis during streptomycete development. When *Streptomyces* spp. had a 24-h or 72-h advantage, various zones inhibiting the growth of fungi were observed after an additional 24 h or 72 h, a condition that may reflect a natural event when a streptomycete inhabits a symbiotic organism and protects it from fungal pathogens [106,107,108,109,110]. Thus, cultures of *Streptomyces* spp. with *C. albicans* revealed inhibitory zones of the yeast that vary between 3 and 12 mm among various, mostly tetrafungin/tetrin A/polyene B-producing streptomycete species. *Aspergillus fumigatus* looked highly resistant to the cytolytic metabolites produced by our set of streptomycetes. *Aspergillus niger*, on the other hand, could not grow in the vicinity of most of the streptomycetes. *Fusarium* spp. was sensitive only to several tetrafungin/tetrin A/polyene B-producing strains, whereas the sole polyene B-producers did not inhibit its growth. Nevertheless, all hemolytic streptomycetes, regardless of their compound production, were more or less able to inhibit the growth of *Paecilomyces* spp. which seemed to be most sensitive of the micromycetes tested.

Our LC-MS data combined with the UV-VIS absorption possibly revealed production of six previously characterized cyclic polyenes: candicidins A1, A2, filipin III, pentamycin (fungichromin), strevertene A, tetrafungin, and tetrin A [24,26,33,34,42,51], and four novel polyenes (A-D) in our set of *Streptomyces* species. Only four strains of the 23 assayed did not show any detectable putative polyenes. This may or may not necessarily mean that another non-polyenic cytotoxic SM is produced. In case of an undetected polyene production, the compounds can be unstable or they are not produced in the amount needed for the isolation method used in this study. Besides this, our results suggest that the production of polyenes is widespread among streptomycetes and often associated with their β-hemolytic and/or antifungal activities.

Tetraenes produced by seven strains belonged to two structural types: small tetrins with MW < 700 [26] and large nystatin and amphotericin B ressembling tetrafungin of MW > 1000 [24]. Unlike tetrins, the structure of tetrafungin in unknown, though the compound was discovered almost 40 years ago. Antifungal activities of the tetraenes have already been reported; however, their hemolytic capabilities were not explored.

Pentaenes were produced by the TR1341 control strain (filipin III and pentamycin). Filipin III was also found in the BCCO 10_2155 strain; however, the associated pentamycin was missing there. The hemolytic properties of the filipin-type compounds, produced by multiple streptomycete species, have long been recognized [111], as well as their antifungal activity. They exhibit equal affinity to ergosterol (the main fungal sterol) and to mammalian cholesterol, which complicates their wider medical use. The sterol-binding feature leads to perforations of the erythrocyte membranes explaining their hemolytic properties [90]. Activity of the filipin gene cluster is essential for β-hemolysis of the human-associated *Streptomyces* sp. TR1341 [89]. Filipin production does not seem to be linked to any particular phylogenetic clade. On the other hand, strevertene A, a similar pentaene lacking the methylated side chain, was found to be produced by two soil strains belonging to the same phylogenetic clade of *S. avidinii* NBRC13426, only (Figure 1). Another producer of strevertenes, the tomato-associated *S. psammoticus,* uses the compound to protect the host plants against *Fusarium* wilt [112]. Of the novel compounds, polyene B shared by 10 strains, shows the typical methylpentaene-specific absorption spectrum as filipin, but has substantially higher molecular mass. The compound is typically found in strains putatively producing tetraenes (Table S2). It seems unusual for a single strain to produce macrolides differing in the ring lengths as this is determined by the type I polyketide synthase (PKS-I) structure. Of course, we cannot exclude the presence of two different biosynthetic gene clusters in the producer strains, or PKS-I flexibility in the starter unit selection/the chain length control, as possible explanations. Next, a possibility of non-enzymatic degradation (e.g., oxidation) of the original, enzymatically synthesized compounds have to be taken in account as documented in nystatin [113]. However, a more detailed genomic analysis is needed to better understand the biosynthesis of these molecules.

Two novel compounds seem to belong to methylhexaenes—if we assume that a methyl group adjacent to the polyene chain shifts the absorption spectrum peaks to slightly higher wavelengths, as documented for methylpentaenes [42]. Polyene D was produced in a single strain pure culture (BCCO 10_2179), whereas polyene C in the culture of BCCO 10_1331 and BCCO 10_2309 strains. Hexaenes are the smallest, most under characterized polyene group, with just one well-described member: linearmycin [67]. The production of a hexaene derivative of nystatin as a result of a spontaneous mutation of the relevant PKS genes in *S. noursei* has also been reported [114]. All other hexaenic compounds have been detected based solely on their characteristic UV-VIS absorption and their antifungal activity (Table 1) and the relevant reports lack detailed structure and activity data. Therefore, the two potential novel hexaenes represent perfect targets for future purification as well as structure and activity assessments.

Heptaenes were represented by candicidins detected in two strains coming from different phylogenetic clades (Figure 1). Candicidins have been first identified in *S. griseus* IMRU3570 [115]. The candicidin complex consists of up to nine compounds (A-I) where only candicidin D has been fully structurally characterized [116]. Candicidins change permeability in the cell membrane of *C. albicans* [117], followed by the release of K+ ions from the intracellular space, which completes the cell lysis [118].

The mass spectrum of the polyene A peak (BCCO 10_1106) was indeterminate and we were therefore not able to determine its putative molecular formula. Its absorption spectrum resembles that of pentaenes, but the maxima are slightly lower. This suggests that the compound’s polyketide backbone might be further modified in an atypical way.

Streptomycetes undergo a complex life cycle that requires highly coordinated gene expression responsive to environmental changes [119,120,121]. Various small-molecular-weight signaling molecules participate in gene expression control. In this manner, GBLs stimulate metabolic production and act as natural auto-regulators [122]. They mediate intra- and/or interspecies communication via the so-called quorum sensing system. They also help them react effectively to competitive organisms as they may accelerate the transition from the exponential growth phase to sporulation, which includes the coordinated production of bioactive compounds [123]. Here we showed a positive effect of GBL on the production of hemolytic metabolites in several strains (Figure S2).

Co-cultures have been used successfully between various streptomycete species to activate secondary metabolism [124]. Likewise, complex hemolytic properties can be changed (increased or decreased) as a result of interaction among different species in one habitat [125]. In this work we applied the co-culture technique to those strains for which we had not been able to detect any hemolytic compound in a standard one-strain cultivation. Despite probable considerable losses of hemolytic compounds from samples when using the SPE “miniprep” technique, we were able to detect new compounds in the paired co-cultures of [BCCO 10_1331+BCCO 10_2309] and [BCCO 10_2259+BCCO 10_2295] strains. The first pair revealed the novel polyene C compound (mentioned above). The second pair revealed a known non-polyenic hemolytic compound actiphenol, a phenol metabolite with antifungal effects [126].

## 4. Materials and Methods

### 4.1. Strains

In total, 23 β-hemolytic isolates (besides one *Nocardioides* spp. they were exclusively strains of *Streptomyces*) were obtained from the Collection of Actinomycetes (Biology Centre Collection of Organisms, BCCO, České Budějovice, the Czech Republic, www.actinomycetes.bcco.cz, 9 November 2022) to compare the hemolytic activities of streptomycete isolates originating from various niches in different regions around the world (the strains are listed in Table S2). Of these, 12 originated from soil, 10 were arthropod-associated, and 1 was plant-associated. One additional strain is the human-associated *Streptomyces* spp. TR1341, a filipin producer that has already been analyzed [89]. This strain was used here as a positive control for the LC-MS analyses.

### 4.2. 16S rRNA-encoding Gene-Based Phylogeny

We constructed a phylogenetic tree in which we indicated the origin of the strain, the source of isolation, and the type of substance produced to determine the relatedness of the strains in this study and to compare the studied strains with known polyene antibiotic producers. The strains belonging to the BCCO collection are characterized using a combination of morphological features and basic molecular identification. The morphological characterization was performed according to the protocols of the International Streptomyces Project [127]. The molecular identification was performed using 16S rRNA gene sequencing and an identity cut-off of 98.7% for classification as a known species [128]. Data are provided in the catalogue of the BCCO web pages (see above, Section 4.1) under the respective strain number. Overall, 113 sequences were obtained and used for the construction of the tree as described elsewhere [129]; 32 sequences belong to strains in the BCCO collection and 24 sequences belong to known polyene producers that are listed in Table S1. The remaining ones are reference strains used for phylogenetic placement. Of the 24 sequences of known polyene producers, 15 were downloaded from the EzTaxon (access on 4 April 2022) [130]. The 57 sequences used for phylogenetic placement were obtained from the NCBI database (access 04/04/2022) [131]. The sequences were aligned using Muscle (v3.8.425) [132], and the alignment was manually checked and edited to 1522 final, informative columns. The alignment was then used to produce a 1000-replicated bootstrap consensus maximum likelihood tree using RAxML (v8.2.11) [133]. The 16S rRNA gene sequence of the *Amycolatopsis alba* DSM44262 was used as outgroup. The tree was edited for publication using iTOL [134].

### 4.3. Culture Media and Cultivation Conditions

For liquid cultures, *Streptomyces* strains were cultivated in 5 mL of GYM medium [135] at 28 °C for 96 h if not stated otherwise. If required, the medium was supplemented with defibrinated rabbit’s blood (7%). Paired co-cultures, i.e., two *Streptomyces* strains inoculated equally into one medium, were performed for those strains in which hemolytic SMs had not been identified from previous one-strain cultures. For hemolytic assays, Columbia sheep blood agar (OXOID CZ s.r.o., Thermo Fisher Scientific, Brno, Czechia) was used.

### 4.4. Ethyl Acetate Extraction and Hemolytic Activity Testing

After centrifugation at 10,000× *g* at 4 °C for 10 min, NaCl was added to the supernatant, up to a concentration of 5 M. Subsequently, 1 mL of ethyl acetate was added to each vial and incubated on a shaker at 250 rpm and 4 °C for 30 min. Samples were centrifuged at 5000× *g* for 10 min, and the upper layer of ethyl acetate was transferred to a rotary vacuum evaporator until the sample was completely dry, followed by the addition of 10 μL of chloroform to each sample. Subsequently, 5 μL of each sample was dropped on a blood agar plate and incubated for 3 days with 5 μL of pure chloroform in the middle as a negative control.

### 4.5. Solid-Phase Extraction

The solid-phase extraction (SPE) procedure was performed as described elsewhere [136]. Briefly, each strain’s supernatant was isolated by centrifugation at 10,000× *g* at 4 °C for 10 min, and the pH was adjusted to 3–4 using formic acid (Merck, Darmstadt, Germany). An Oasis HLB 3cc 60 mg cartridge (hydrophilic-lipophilic balanced sorbent, Waters, Milford, MA, USA) was conditioned with 3 mL methanol, equilibrated with 3 mL Milli-Q water (Sigma-Aldrich Co. St. Louis, MO, USA), and subsequently, 3 mL of culture supernatant was loaded. The cartridge was then washed with 3 mL of water, and the absorbed substances were eluted with 1.5 mL of methanol.

### 4.6. LC-MS Analysis

The eluent of each strain was evaporated to dryness (Concentrator plus/Vacufuge^®^ plus, Eppendorf AG, Hamburg, Germany), and 150 μL of 50% methanol was added to each vial. The vials were centrifuged for 5 min at 5000 rpm (Centrifuge MiniSpin, Rotor F-45-12-11, Eppendorf AG, Hamburg, Germany) and 50 µL of each sample was then loaded onto the LC-MS. 

The analyses were performed on the Acquity UPLC system with a 2996 PDA detection system (194–600 nm), connected to an LCT premier XE time-of-flight mass spectrometer (Waters, Milford, MA, USA). A 5 μL aliquot of each sample was loaded onto the Acquity UPLC BEH C18 LC column (50 mm × 2.1 mm I.D., particle size 1.7 μm, Waters, Milford, MA, USA), kept at 40 °C, and eluted with a two-component mobile phase, A and B, consisting of 0.1% formic acid and acetonitrile, respectively, at a flow rate of 0.4 mL min^−1^. The analyses were performed under a linear gradient program (min/%B) 0/5; 1.5/5; 15/70; 18/99 followed by a 1.0-min column clean-up (99% B) and 1.5-min equilibration (5% B). The mass spectrometer operated in the positive “W” mode with capillary voltage set at +2800 V, cone voltage +40 V, dissolving gas temperature: 350 °C, ion source block temperature, 120 °C, cone gas flow 50 L h^−1^, dissolving gas flow 800 L h^−1^, scan time of 0.15 s, and an inter-scan delay of 0.01 s. The mass accuracy was kept below 6 ppm using the lock spray technology with leucine enkephalin as the reference compound (2 ng μL^−1^, 5 μL min^−1^). The MS chromatograms were extracted for [M+H]^+^ ions with a tolerance window of 0.05 Da, smoothed with the mean smoothing method (window size; four scans, number of smooths, two). The data were processed by MassLynx V4.1 (Waters, Milford, MA, USA). The original method is described elsewhere [137].

### 4.7. Testing for the Presence of Extracellular Hemolytic Proteins

To test for the presence of extracellular hemolytic proteins [138], the supernatant collected (cultured 72 h, 28 °C, 200 rpm, centrifuged at 4000× *g*, 10 min) was filtered (pore size 5 microns) and divided into thirds. The first third of the supernatant was incubated with proteinase K (Carl Roth, Karlsruhe, Germany) to a final concentration of 1 μg/mL at 37 °C for 60 min. The second third of the supernatant was incubated under the same conditions but without the enzyme (this sample served as a positive control as it was expected to maintain its hemolytic activity). The third part was incubated at 100 °C for 5 min (a negative control as we expected a loss of any hemolytic activity). We applied 5 μL of each sample to a paper disc placed on the blood agar. The samples were cultured overnight at 28 °C.

### 4.8. Antifungal Activity of Streptomyces

Selected *Streptomyces* strains were inoculated into a 1cm^2^ square shape in the middle of a blood agar Petri dish and cultivated at 28° C. After 0, 24 h, and 72 h, clinical isolates of *Aspergillus niger, Aspergillus fumigatus, Fusarium* spp., and *Paecilomyces* spp. were added in lines leading to the *Streptomyces* square and co-cultured at the same conditions for another 72 h. The growth inhibitory zones of the fungi were then measured.

## 5. Conclusions

An extensive literature review on polyenes and non-polyene hemolytic compounds produced by streptomycetes is presented. Among the secondary metabolites produced by a set of 23 β-hemolytic *Streptomyces* strains, known—*candicidins, filipins, strevertene A, tetrafungin*, and *tetrin A*—and novel—*polyene AD*—polyenic compounds were found. The new compound producers were incorporated into a streptomycete phylogenetic tree among known polyene producers. No clear relation between phylogeny and production of specific polyene types could be revealed. The obtained results suggested that SMs are responsible for hemolytic activities in *Streptomyces* spp. Their production was in some cases inducible by GBL or by co-cultivation with other *Streptomyces* strains. Our data suggest that streptomycetes may still serve as a promising source of novel SMs with antifungal and/or hemolytic activities.

## Figures and Tables

**Table 1:** Actinomycete polyene SMs. AB—antibacterial; AF—antifungal; AP/I—antiparasitic, insecticidal; HL/CL—hemo-/cytolytic; AC—anti-cancer. Asterisks indicate compounds with clinical (*) or agricultural (**) application.

Compound	Formula	Calculated Average Mass	Activities					Ref.
**CYCLIC—TETRAENES**			**AB**	**AF**	**AP/I**	**HL/CL**	**AC**	
Amphotericin A *	C_47_H_75_NO_17_	926.1090		+		+		[13]
Antifungalmycin 702	C_35_H_60_O_14_	704.8530		+				[14]
Arenomycin B (Lucensomycin)	C_36_H_55_NO_13_	709.8316		+				[15]
Aureofuscin	C_28_H_43_NO_12_	585.6490		+				[16]
Lucimycin (Lucensomycin, Etruscomycin)	C_36_H_53_NO_13_	707.8158		+		+		[17]
NPP A1	C_55_H_88_N_2_O_22_	1129.3040		+				[18]
Nystatin A1 (Fungicidin) *	C_47_H_75_NO_17_	926.1090	-	+				[19]
Nystatin A2	C_47_H_75_NO_16_	910.1096						
Nystatin A3	C_53_H_85_NO_20_	1056.2526						
Pimaricin (Natamycin) */**	C_33_H_47_NO_13_	665.7351		+		-		[20]
Polyfungin B	C_53_H_85_NO_19_	1040.2532		+				[21]
Protocidin	C_29_H_45_NO_13_	615.6752						[22]
Rimocidin	C_39_H_61_NO_14_	767.9117	-	+				[23]
Tetrafungin	C_47_H_82_NO_23_	1029.1610		+				[24]
Tetramycin A Tetramycin B	C_35_H_53_NO_13_ C_35_H_53_NO_14_	695.8048 711.8042		+	+			[25]

Tetrin A Tetrin B Tetrin C	C_34_H_51_NO_13_ C_34_H_51_NO_14_ C_34_H_49_NO_13_	681.7779 697.7773 679.7620		+				[26]
								[27]
Toyamycin (Akitamycin)	C_41_H_65_NO_18_	859.9630		+				[28]
PA-166	C_35_H_53_NO_14_	711.8042		+				[28]
**CYCLIC—PENTAENES**			**AB**	**AF**	**AP/I**	**HL/CL**	**AC**	
Aurenin (1’-Hydroxyisochainin)	C_33_H_54_O_11_	626.7852		+				[29]
Capacidin	C_54_H_85_NO_18_	1050.2716		+				[30]
Chainin	C_33_H_54_O_10_	610.7858		+				[31]
Elizabethin	C_35_H_58_O_12_	670.8383		+				[32]
Filipin I	C_35_H_58_O_9_	622.8401		+		+		[33]
Filipin II	C_35_H_58_O_10_	638.8395						
Filipin III	C_35_H_58_O_11_	654.8389						
Filipin IV	C_35_H_58_O_11_	654.8389						
Fungichromin (Pentamycin) *	C_35_H_58_O_12_	670.8383	+	+		+		[34]
Homochainin	C_34_H_56_O_10_	624.8126		+				[31]
Isochainin	C_33_H_54_O_10_	610.7858		+				[35]
Kabicidin	C_35_H_60_O_13_	688.8536		+				[36]
Lienomycin	C_67_H_107_NO_18_	1214.5825	+	+			+	[37]
Moldicidin A	C_42_H_81_NO_19_	904.1005		+				[16]
Norchainin	C_32_H_52_O_10_	596.7589		+				[31]
Onomycin-I Onomycin-II	C_43_H_76_NO_17_ C_42_H_67_NO_17_	879.0730 857.9905		+				[38]
Pentacidin	C_31_H_50_O_10_	582.7320		+				[16]
Pentafungin	C_41_H_74_NO_16_	837.0357		+				[39]
PA-153	C_37_H_61_NO_14_	743.8897		+				[28]
S 728	C_56_H_93_NO_20_	1100.3492		+				[16]
Reedsmycin A-E Reedsmycin F	C_36_H_58_O_10_ C_36_H_58_O_11_	650.8505 666.8499		+				[40]
Selvamicin	C_47_H_76_O_18_	929,0955		+				[41]
Strevertene A Strevertene B Strevertene C Strevertene D Strevertene E Strevertene F Strevertene G	C_31_H_48_O_10_ C_32_H_50_O_10_ C_32_H_50_O_10_ C_33_H_52_O_10_ C_33_H_52_O_10_ C_34_H_54_O_10_ C_31_H_50_O_9_	580.7161 594.7430 594.7430 608.7699 608.7699 622.7968 566.7326		+				[42]
Takanawaene A Takanawaene B Takanawaene C	C_30_H_48_O_8_ C_32_H_52_O_8_ C_33_H_51_O_8_	536.7063 564.7601 578.7870		+				[43]
Thailandin A Thailandin B	C_39_H_62_O_14_ C_33_H_52_O_10_	754.9208 608.7699		+				[44]
**CYCLIC—HEXAENES**			**AB**	**AF**	**AP/I**	**HL/CL**	**AC**	
Candihexin A	C_48_H_76_NO_19_/C_43_H_76_NO_19_/C_43_H_77_NO_19_	971.1268/911.0718/912.0797		+				[45]
Candihexin B	C_48_H_90_NO_21_/C_48_H_91_NO_21_	1017.2367/1018.2447		+				[45]
Candihexin E	C_38_H_67_NO_16_	793.9471		+				[45]
Cryptocidin	C_52_H_84_NO_17_	995.2355	+	+				[46]
Grecomycin	C_38_H_41_O_10_/C_38_H_38_O_10_	657.7375/654.712	+	+				[47]
**CYCLIC—HEPTAENES**			**AB**	**AF**	**AP/I**	**HL/CL**	**AC**	
Acmycin	C_36_H_68_NO_30_	994.9247		+				[48]
Amphotericin B	C_47_H_73_NO_17_	924.0932		+	+			[49]
Aureofungin A	C_59_H_86_N_2_O_19_/C_59_H_88_N_2_O_19_	1127.3339/1129.3498		+				[50]
Aureofungin B	C_57_H_85_NO_19_/C_57_H_87_NO_19_	1088.2972/1090.3131		+				[50]
Candicidin A1 (VI, Levorin A0, Ascosin A1) * Candicidin A2 (D, A, D1, Levorin A2, Ascosin A2) Candicidin A3 (V, Levorin A3, Ascosin A3)	C_59_H_84_N_2_O_17_ C_59_H_84_N_2_O_18_ C_59_H_86_N_2_O_18_	1093.3272 1109.3186 1111.3345		+		+		[51]
Candidin	C_47_H_71_NO_17_	922.0773		+				[52]
Flavumycin A	C_60_H_91_N_2_O_17_/C_54_H_79_NO_16_	1112.3858/998.2184		+				[53]
Fungimycin	C_59_H_86_N_2_O_17_	1095.3351		+	+	+		[54]
Hamycin A	C_58_H_86_N_2_O_19_	1115.3229		+	+	+		[55]
Isolevorin A2	C_60_H_86_N_2_O_18_	1123.3455		+				[56]
Levorin A2	C_59_H_86_N_2_O_18_/C_59_H_89_N_2_O_18_	1111.3345/1114.3583		+				[57]
Levorin B	C_62_H_98_N_2_O_25_	1271.4586		+				[58]
Lucknomycin	C_61_H_98_N_2_O_24_/C_54_H_80_N_2_O_19_	1243.4482/1061.2313		+				[59]
Partricin A	C_59_H_86_N_2_O_9_	967.3399		+				[60]
Partricin B	C_55_H_84_N_2_O_19_	1077.2740		+				[60]
Perimycin A	C_59_H_88_N_2_O_17_	1097.3510		+				[61]
Trichomycin A	C_58_H_84_N_2_O_18_/C_61_H_86_N_2_O_21_	1097.3076/1183.3547		+	+			[62]
AF-1231	C_42_H_68_N_2_O_17_	873.0052		+				[16]
DJ-400 B_1_ DJ-400 B_2_	C_65_H_96_N_2_O_21_ C_58_H_86_N_2_O_20_	1241.4781 1131.3223		+				[38]
67-121 A 67-121 C	C_59_H_88_N_2_O_19_ C_65_H_98_N_2_O_28_	1129.3498 1355.4898		+				[38]
NPP B1	C_55_H_86_N_2_O_22_	1127.2881		+				[18]
**LINEAR POLYENES**			**AB**	**AF**	**AP/I**	**HL/CL**	**AC**	
AB023a AB023b	C_31_H_50_O_8_ C_32_H_52_O_8_	550.7332 564.7601		+				[63]
Clethramycin	C_63_H_99_N_3_O_18_S	1218.5545		+				[64]
ECO-02301	C_70_H_109_N_2_O_20_	1298.6369		+				[65]
Etnangien	C_49_H_76_O_11_	841.1358	+					[66]
Linearmycin A Linearmycin B Linearmycin C	C_64_H_101_NO_16_ C_66_H_103_NO_16_ C_67_H_105_NO_16_	1140.5031 1166.5410 1180.5678	+	+		+		[67]
Mediomycin Mediomycin A Mediomycin B	C_62_H_99_NO_16_S C_62_H_97_NO_18_S C_62_H_97_NO_15_	1146.5312 1176.5141 1096.4499		+				[64]
Meijiemycin	C_66_H_105_NO_19_	1216.5550		+				[68]
Mycangimycin	C_20_H_24_O_4_	328.4082		+				[69]
Neotetrafibricin A	C_67_H_105_NO_19_	1228.5660		+				[64]

**Table 2:** Non-polyene SMs of actinomycetes targeting human cells.

Compound	Formula	Calculated Average Mass	Activities	Ref.
**PEPTIDES**				
Surugamide A	C_48_H_81_N_9_O_8_	912.21428	anticancer, antifungal	[70]
Polyoxypeptin	C_35_H_60_O_14_	704.84403	pro-apoptotic	[71]
Bleomycin	C_55_H_84_N_17_O_21_S_3_+	1415.55415	anti-cancer	[72]
Actinomycin D	C_62_H_86_N_12_O_16_	1255.41969	anti-cancer	[73]
Mirubactin	C_26_H_32_N_6_O_11_	604.56701	siderophore	[74]
**ANTIMYCINS**				
Antimycin A	C_24_H_40_N_2_O_9_	548.62641	inhibitor of respiration	[75]
**NON-POLYENIC MACROLIDES**				
FK506 (Tacrolimus)	C_44_H_69_NO_12_	804.02005	immunosuppressive, antifungal	[76]
FK520 (Ascomycin)	C_43_H_69_NO_12_	792.00931	immunosuppressive, antifungal	[77]
Meridamycin	C_45_H_75_NO_12_	822.07844	neuroprotective	[78]
Nemadectin	C_36_H_52_O_8_	612.79481	antiparasitic	[79]
Sirolimus (Rapamycin)–a cyclic molecule containing conjugated triene.	C_51_H_79_NO_13_	914.17404	immunosuppressive, antifungal	[80]
Venturicidin B	C_40_H_66_O_10_	706.94776	antifungal	[81]
**NON-POLYENIC POLYKETIDES**				
Actiphenol	C_15_H_17_NO_4_	275.30042	proteosynthesis inhibitor	[82]
Kinamycin F	C_18_H_14_N_2_O_7_	370.31373	anti-cancer	[83]
Neoansamycin A	C_30_H_37_NO_7_	523.61855	antibiotic, antiviral	[84]
Nogalamycin	C_39_H_49_NO_16_	787.80515	anti-cancer	[85]
Reveromycin A	C_36_H_52_O_11_	660.79303	EGF inhibitor	[86]
**OTHER—ACTIVE ON THE HUMAN CELLS**				
Neocarzinostatin	C_35_H_33_NO_12_	659.63751	anti-cancer	[87]
Nocardamine	C_27_H_48_N_6_O_9_	600.70599	anti-cancer siderophore	[88]

**Table 3:** Inhibitory activity of *Streptomyces* spp. against *Candida albicans*.

BCCO Strain No.	Metabolite Produced	*Streptomyces* sp. (Vertical Line) *Candida albicans* (Horizontal Line)	Size of Inhibitory Zone (mm)	Size of Hemolytic Zone (mm)
10_1099	candicidin A, A1, A3; surugamide A	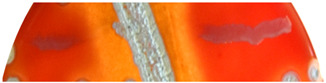	10	7
10_1093	tetrafungin, tetrin A, polyene B	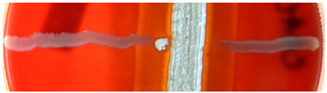	5	9
10_1094	tetrafungin, tetrin A, polyene B	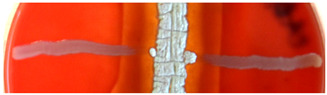	3	9
10_1095	tetrafungin, tetrin A, polyene B	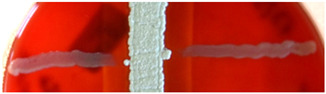	3	7
10_1104	tetrafungin, tetrin A, polyene B	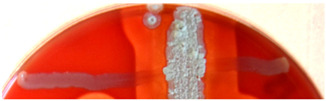	8	8
10_2282	tetrafungin, tetrin A, polyene B	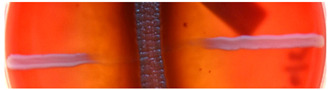	12	16

**Table 4:** Inhibitory activity of *Streptomyces* spp. against filamentous fungi. Streptomycetes were inoculated in a square shape and the fungi were inoculated in lines. The size of the observed inhibitory zones is presented in mm above each picture. Pictures of hemolytic zones of streptomycetes were taken from the bottom of the dishes.

BCCO Strain No./Found Metabolite	*Paecilomyces* spp.	*Fusarium* spp.	*Aspergillus fumigatus*	*Aspergillus niger*	Cultivation Timing/Streptomycete Hemolysis
10_2259	6	0	0	0	Streptomyces 144 h; fungus 72 h
actiphenol (?)	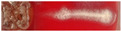	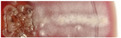	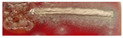	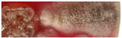	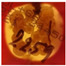
10_1099	10	no growth	0	6	Streptomyces 96 h; fungus 72h
candicidin	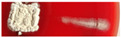	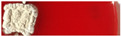	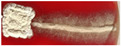	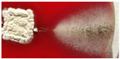	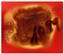
10_1106	8	0	0	2	Streptomyces 96 h; fungus 72 h
polyene A	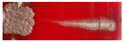	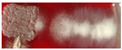	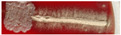	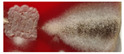	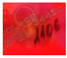
10_1092	11	no growth	3	8	Streptomyces 144 h; fungus 72 h
tetrafungin, tetrin A, polyene B	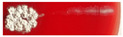	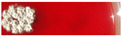	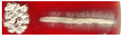	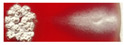	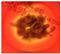
10_1093	8	no growth	1	6	Streptomyces 96 h; fungus 72 h
tetrafungin, tetrin A, polyene B	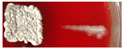	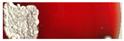	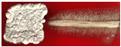	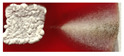	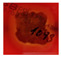
10_1094	20	no growth	1	5	Streptomyces 96 h; fungus 72 h
tetrafungin, tetrin A, polyene B	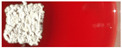	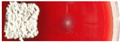	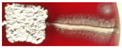	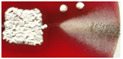	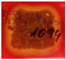
10_1095	5	0	0	4	Streptomyces 96 h; fungus 72 h
tetrafungin, tetrin A, polyene B	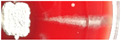	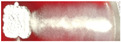	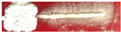	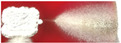	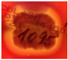
10_1097	12	0	0	9	Streptomyces 96 h; fungus 72 h
polyene B	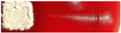	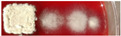	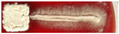	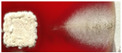	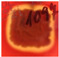
10_1104	24	3	3	16	Streptomyces 144 h; fungus 72 h
tetrafungin, tetrin A, polyene B	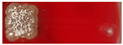	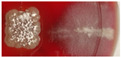	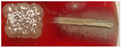	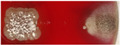	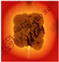
10_2282	20	0	0	6	Streptomyces 144 h; fungus 72 h
tetrafungin, tetrin A, polyene B	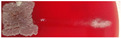	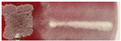	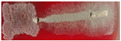	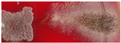	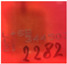
10_2389	no growth	0	0	12	Streptomyces 144 h; fungus 72 h
polyene B	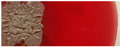	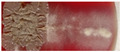	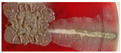	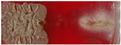	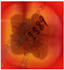
10_2309	24	0	0	4	Streptomyces 144 h; fungus 72 h
polyene C (?)	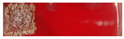	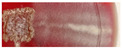	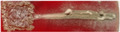	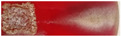	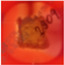

## Data Availability

Not applicable.

## Supplementary Materials

The following supporting information can be downloaded at: https://www.mdpi.com/article/10.3390/ijms232315045/s1.
