# Supplementary figures and images for: Polyenic Antibiotics and Other Antifungal Compounds Produced by Hemolytic Streptomyces Species
Source: Int J Mol Sci. 2022 Nov 30;23(23):15045. doi: 10.3390/ijms232315045 (PMC9740855; doi:10.3390/ijms232315045)

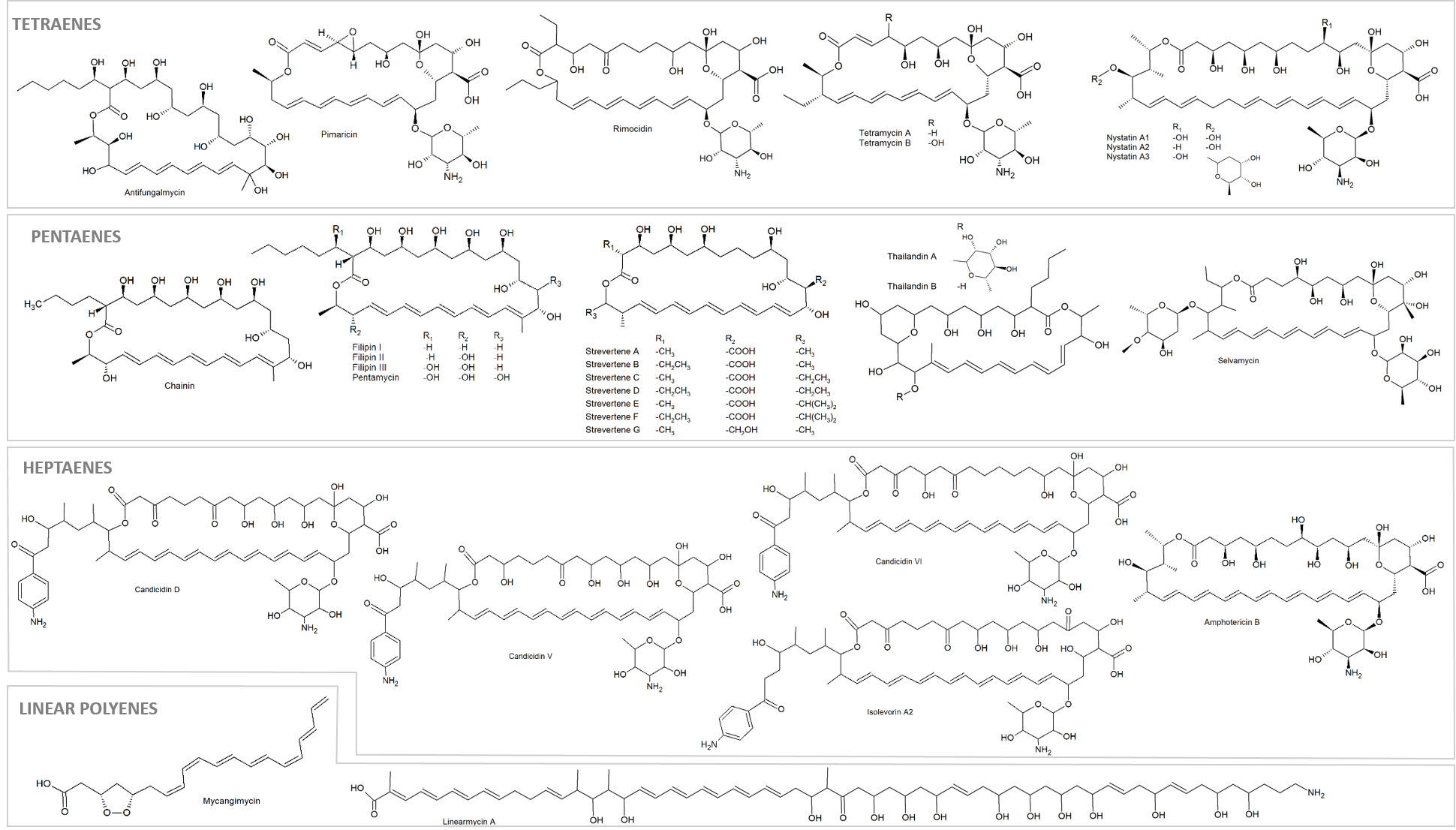

Supplement: Supplementary file 1 [file ijms-23-15045-s001.zip › Figure S1 Polyenes Formulas.png]

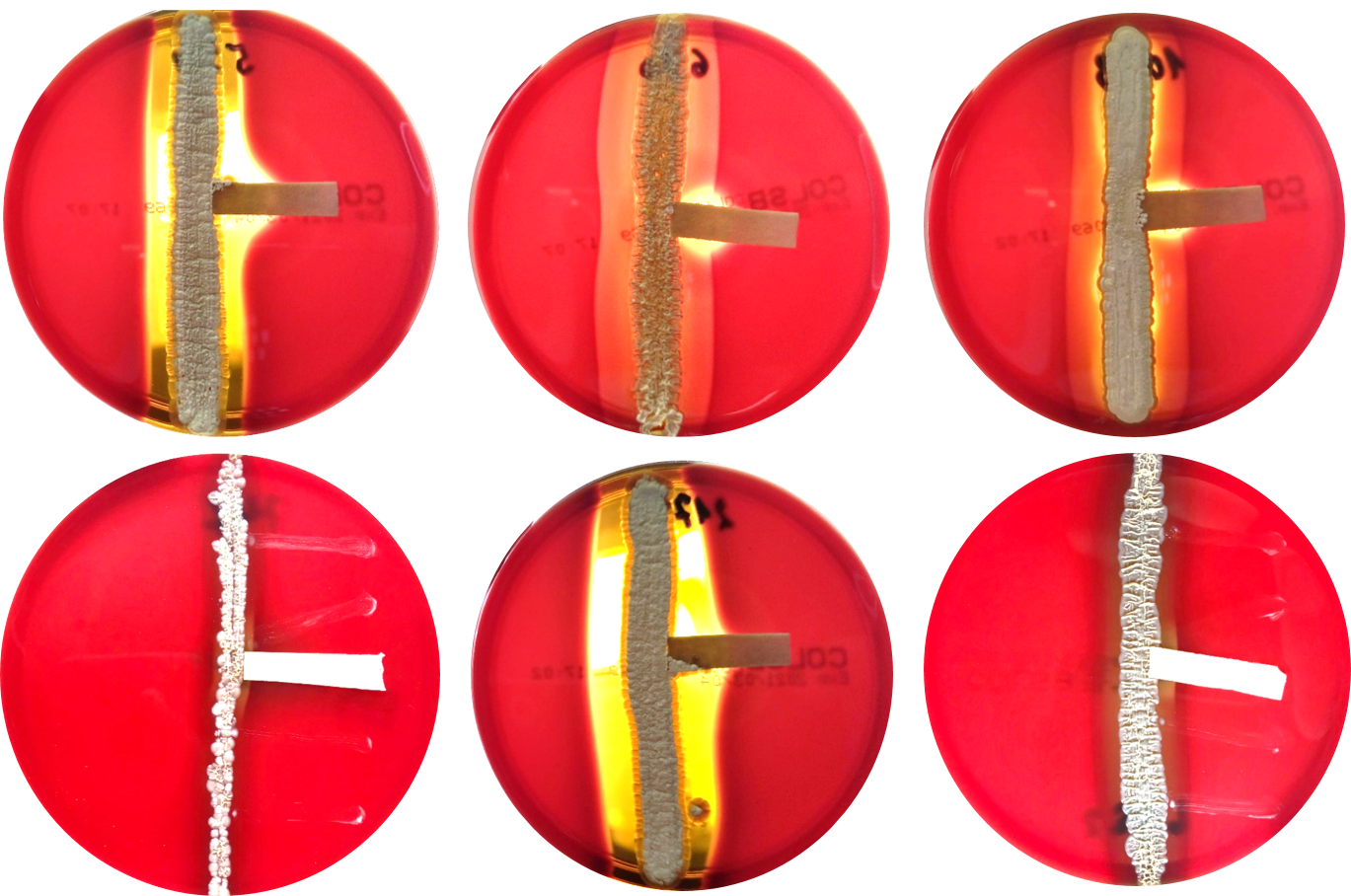

Supplement: Supplementary file 1 [file ijms-23-15045-s001.zip › Figure S2. GBL-induced hemolytic activity in Streptomyces strains.png]

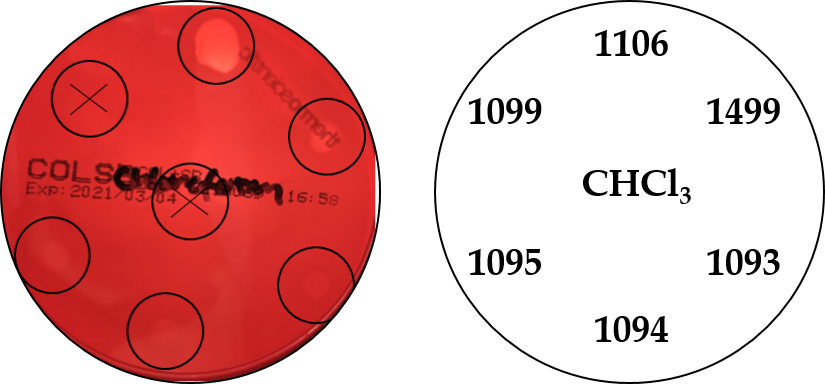

Supplement: Supplementary file 1 [file ijms-23-15045-s001.zip › Figure S3. Hemolytic activity of ethyl acetate extracts.png]

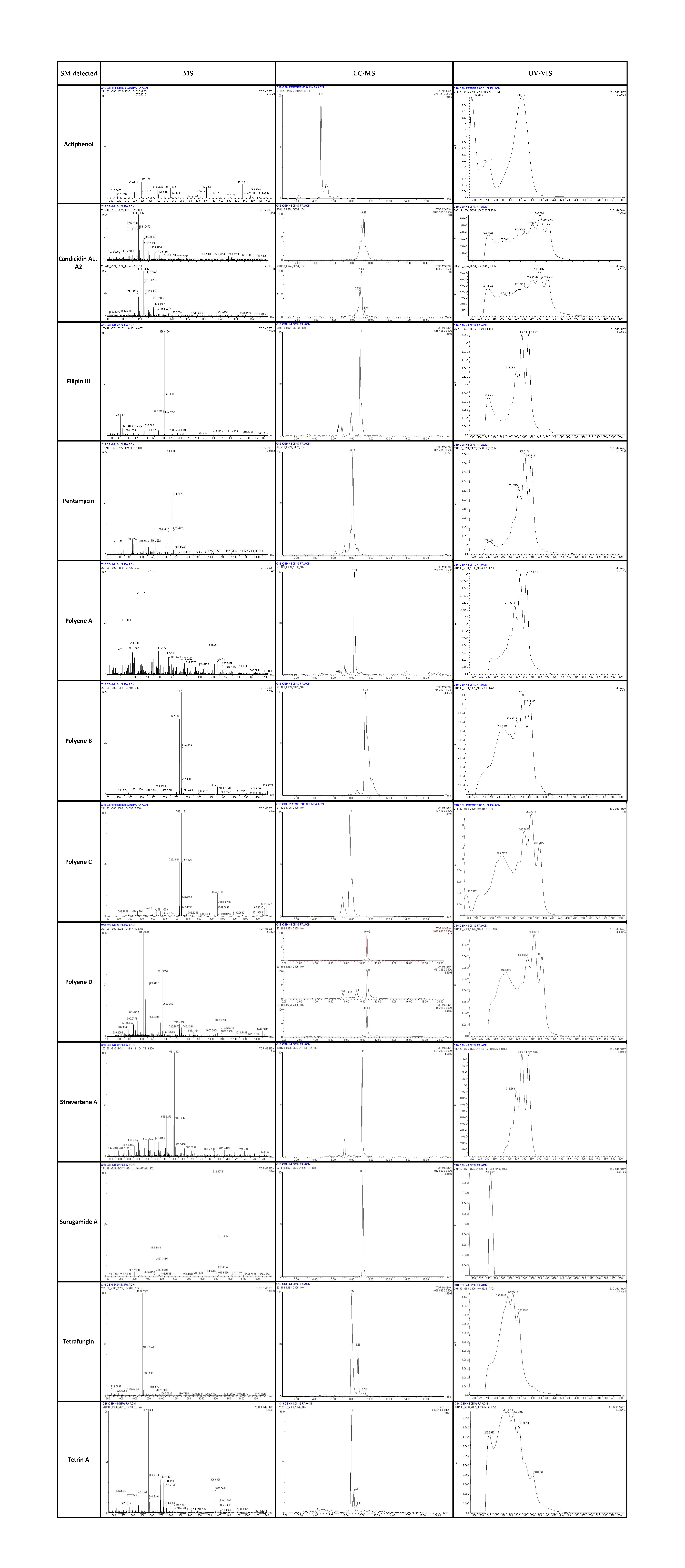

Supplement: Supplementary file 1 [file ijms-23-15045-s001.zip › Table S3 - Chromatograms.png]
